# The Toughest Material in the Plant Kingdom: An Update on Sporopollenin

**DOI:** 10.3389/fpls.2021.703864

**Published:** 2021-09-03

**Authors:** Etienne Grienenberger, Teagen D. Quilichini

**Affiliations:** ^1^Institut de biologie moléculaire des plantes, CNRS, Université de Strasbourg, Strasbourg, France; ^2^Aquatic and Crop Resource Development Research Centre, National Research Council Canada, Saskatoon, SK, Canada

**Keywords:** sporopollenin, biopolymer, spore, pollen, exine cell wall, polyketide, α-pyrone, molecular structure

## Abstract

The extreme chemical and physical recalcitrance of sporopollenin deems this biopolymer among the most resilient organic materials on Earth. As the primary material fortifying spore and pollen cell walls, sporopollenin is touted as a critical innovation in the progression of plant life to a terrestrial setting. Although crucial for its protective role in plant reproduction, the inert nature of sporopollenin has challenged efforts to determine its composition for decades. Revised structural, chemical, and genetic experimentation efforts have produced dramatic advances in elucidating the molecular structure of this biopolymer and the mechanisms of its synthesis. Bypassing many of the challenges with material fragmentation and solubilization, insights from functional characterizations of sporopollenin biogenesis *in planta*, and *in vitro*, through a gene-targeted approach suggest a backbone of polyhydroxylated polyketide-based subunits and remarkable conservation of biochemical pathways for sporopollenin biosynthesis across the plant kingdom. Recent optimization of solid-state NMR and targeted degradation methods for sporopollenin analysis confirms polyhydroxylated α-pyrone subunits, as well as hydroxylated aliphatic units, and unique cross-linkage heterogeneity. We examine the cross-disciplinary efforts to solve the sporopollenin composition puzzle and illustrate a working model of sporopollenin’s molecular structure and biosynthesis. Emerging controversies and remaining knowledge gaps are discussed, including the degree of aromaticity, cross-linkage profiles, and extent of chemical conservation of sporopollenin among land plants. The recent developments in sporopollenin research present diverse opportunities for harnessing the extraordinary properties of this abundant and stable biomaterial for sustainable microcapsule applications and synthetic material designs.

## Introduction

A seminal innovation in plant evolution was the fortification of mobile reproductive cells with sporopollenin. Amidst novel stresses, reinforcement of spore cell walls with a desiccation-resistant, durable exterior of sporopollenin likely provided an adaptive advantage to plant terrestrial reproduction. Lauded as the “diamond of the plant world,” sporopollenin is an organic material distinguished by its extraordinary stability and recalcitrance when challenged by mechanical, thermal, hydrostatic, non-oxidative chemical, and biological pressures (Kesseler and Harley, 2004; Barrier, 2008; Montgomery et al., 2016). The inert nature of sporopollenin is perhaps best highlighted by its chemical preservation in fossil spores an estimated 450 million years old, providing the earliest record of plant life on land (Brooks and Shaw, 1971; Brown and Lemmon, 2011). The ubiquitous appearance of sporopollenin in the outer shell of land plant spores and pollen grains, and strong evolutionary conservation of genes encoding key enzymes in the biosynthesis of sporopollenin among current land plants, support a critical function for this biopolymer in the conquest and proliferative success of plants on dry land (Wallace et al., 2011; Gómez et al., 2015; Quilichini et al., 2015; Shi et al., 2015).

The definition of sporopollenin as the organic residue remaining after acetolysis conveys the nebulous understanding of this biomacromolecule that has prevailed (Zetzsche and Vicari, 1931; Brown, 1960; Hemsley et al., 2009). This is in stark contrast to other major and more widely known biopolymers that fortify plant cell walls, including cutin, suberin, and lignin, for which chemical composition and linkage data are extensive (Mackenzie et al., 2015). Much of the uncertainty surrounding sporopollenin is due to the material’s extreme inertness, preventing solubilization under harsh treatments and yielding partial, modified or degraded fragments of the native polymer for biochemical characterization (Hemsley et al., 2009; Li et al., 2019a). From early models of a carotenoid-based polymer (Brooks and Shaw, 1968), the tools available for elucidating sporopollenin’s composition have grown rapidly, supporting substantial revision of molecular structure models. Circumventing challenges of sporopollenin solubilization and breakdown, genetic tools predominantly in model flowering plants have uncovered a core set of biosynthetic enzymes required for sporopollenin biosynthesis. Mounting evidence from non-model species supports strong conservation of a sporopollenin metabolic pathway across the plant kingdom. Mutant analyses, *in vitro* enzyme assays, labeling studies, and chemical breakdown analyses have facilitated informed inferences on sporopollenin’s composition, supporting a polyhydroxylated aliphatic backbone of polyketides, an aromatic fraction, and extensive coupling of subunits through ester and ether cross-linkages (Quilichini et al., 2015). Until recently however, genetic studies could not fully explain models from biochemical analyses and vice versa.

A recent surge in the analytical characterization of sporopollenin has produced significant breakthroughs in the elucidation of molecular structure, complimenting and challenging prevailing theories. Aided by the development of treatment approaches for interrogating the biopolymer, and the large quantities of spores/pollen available from non-flowering species, mass spectrometry, solid-state NMR, and infrared spectroscopy techniques have uncovered novel structural and compositional data on sporopollenin. Among these discoveries, subunits of varying phobicity coupled by linkages with different solvent stabilities offer plausible explanations for the unique recalcitrance of sporopollenin. While a clearer picture for sporopollenin is emerging, new discrepancies between studies from disparate plant groups and methodologies raise questions about the extent of chemical conservation versus heterogeneity of sporopollenin among land plants. Here, we highlight the latest breakthroughs and emerging controversies in sporopollenin research, and the value of solving the elusive structure of sporopollenin for biotechnological advances.

## Conserved Metabolic Pathways for Sporopollenin Biosynthesis

In flowering plants, sporopollenin is synthesized in tapetal cells that line the inner surface of the anther. Following its synthesis, sporopollenin constituents must exit the tapetum, traverse a fluid-filled locule, and assemble into the structured outer pollen wall or exine surrounding immature pollen grains (or microspores). Although questions surrounding the mechanisms of sporopollenin delivery from the sporophytic tapetum to the developing male gametophyte remain largely unanswered and beyond the scope of this article, evidence from several species supports a role for transport proteins, including ATP-binding cassette transporters and lipid transfer proteins, in sporopollenin export and/or shuttling from the tapetum (Liang et al., 2010; Quilichini et al., 2010, 2014; Choi et al., 2011; Huang et al., 2013; Niu et al., 2013; Qin et al., 2013; Zhu et al., 2013; Zhao et al., 2015; Chang et al., 2016, 2018; Zaidi et al., 2020). Similarly, little is known about the polymerization of sporopollenin, but recent studies implicate reactive oxygen species (ROS) and the environment of the locule in the biopolymer’s assembly (see section entitled “Cross-linkages and Assembly of Sporopollenin” below).

**Figure 1:**
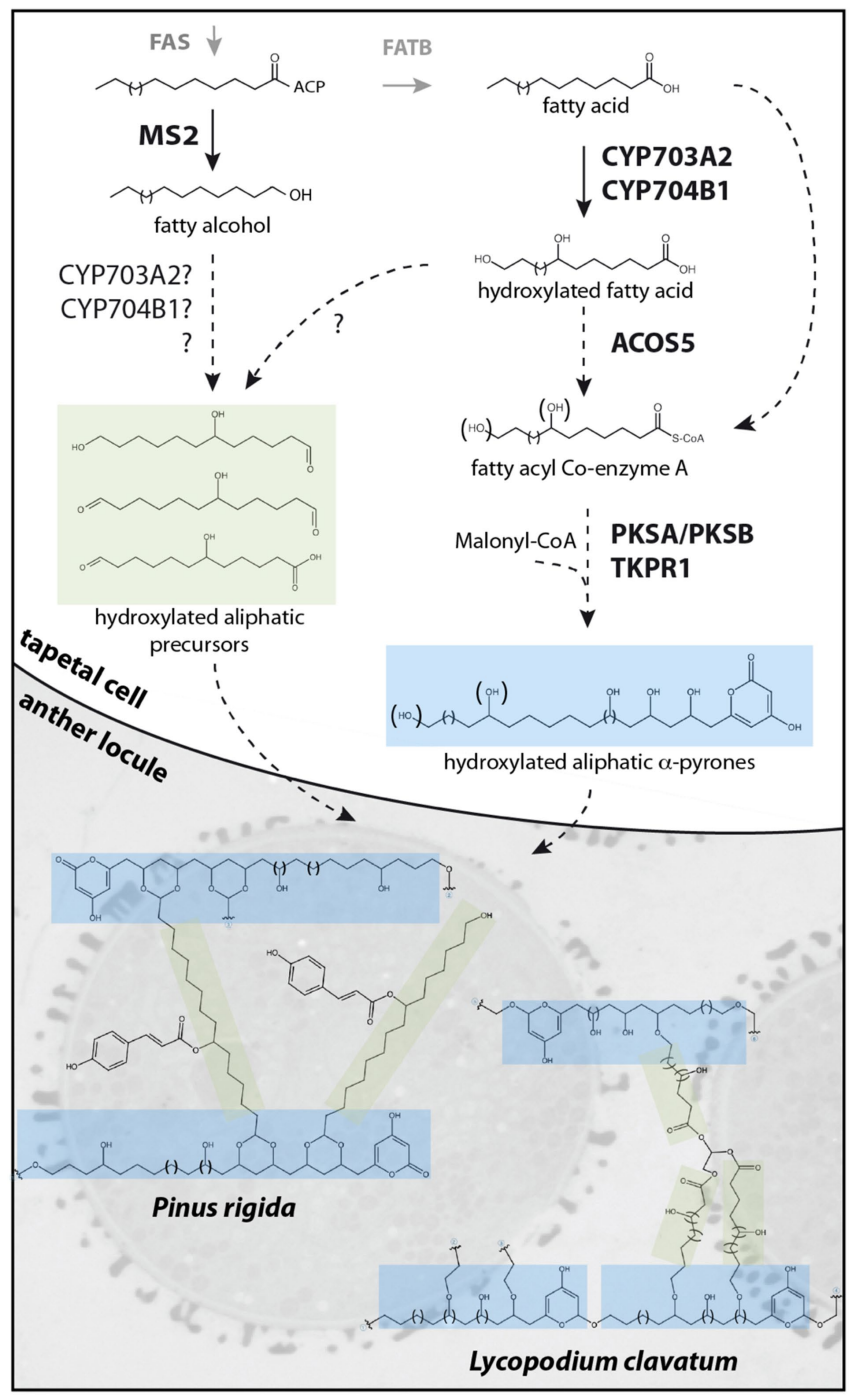
Proposed sporopollenin metabolic pathway and simplified molecular structures of *Pinus rigida* and *Lycopodium clavatum* sporopollenin. Fatty acyl-ACP esters, synthesized de novo by the FAS complex, are reduced by MS2 to produce fatty alcohols or hydrolyzed by FATB to produce free fatty acids. After hydroxylation by CYP703A2 and CYP704B1, free fatty acids may be reduced by unknown reductases to fatty alcohols or aldehydes, forming putative hydroxylated aliphatic sporopollenin precursors. Alternatively, hydroxylation by CYP703A2 and CYP704B1 of the fatty alcohol produced by MS2 may occur. In the case of *Pinus rigida*, these aliphatic precursors might be acylated with phenolics by an unknown transferase. Hydroxylated or non-hydroxylated fatty acids are esterified to CoA by ACOS5 prior to several cycles of condensation by PKSs and reduction by TKPRs to form polyhydroxylated α-pyrone precursors. The precursors could be exported to the anther locule and polymerized on the surface of developing microspores by an unknown mechanism. In *Pinus rigida*, a simplified sporopollenin is proposed to predominantly contain hydroxylated aliphatic α-pyrone units, crosslinked at one end through an ester group and linked through a dioxane moiety to other units by a hydroxylated aliphatic chain bearing a coumaroyl moiety. In *Lycopodium clavatum*, the sporopollenin structure is proposed to contain a rigid macrocyclic backbone of several hydroxylated aliphatic α-pyrone units coupled through ether bonds to hydroxylated aliphatic networks linked together by glycerol.

In contrast to sporopollenin traffic and assembly, more is known about sporopollenin biosynthesis, with a suite of tapetum-expressed genes including *MALE STERILITY2* (*MS2;* Aarts et al., 1997; Chen et al., 2011), *CYTOCHROME P450 (CYP) 703A2* (Morant et al., 2007), *CYP704B1* (Dobritsa et al., 2009), *ACYL-COA SYNTHTASE5* (*ACOS5*; Souza et al., 2009), *POLYKETIDE SYNTHASE A/LESS ADHESIVE POLLEN6* (*PKSA*/*LAP6), PKSB*/*LAP5* (Dobritsa et al., 2010; Kim et al., 2010), and *TETRAKETIDE α-PYRONE REDUCTASE1*/*DIHYDROFLAVONOL 4-REDUCTASE-LIKE1* (*TKPR1*/*DRL1;* Tang et al., 2009; Grienenberger et al., 2010) encoding enzymes required for sporopollenin biosynthesis in *Arabidopsis thaliana* (Arabidopsis; Table 1). Loss-of-function mutations in these genes affect male fertility and disrupt exine deposition, supporting functions for the encoded enzymes in synthesizing crucial sporopollenin constituents. Enzyme assays characterizing recombinant MS2, CYP703A2, CYP704B1, ACOS5, PKSA/LAP6, PKSB/LAP5, and TKPR1/DRL1 support a major role for lipid metabolism in sporopollenin production. *In vitro*, medium- to long-chain fatty acids with varying degrees of hydroxylation are accepted by Arabidopsis ACOS5, forming activated fatty acyl-CoA esters that can be condensed with malonyl-CoAs to form tri- and tetraketide α-pyrones by PKSA/LAP6 and PKSB/LAP5 enzymes (reviewed by Quilichini et al., 2015). Subsequent ketone reduction by TKPR1/DRL1 produces polyhydroxylated aliphatic α-pyrones that are proposed to form core monomers of sporopollenin (Figure 1, blue shading). In addition, the production of fatty alcohols by reduction of long-chain fatty acyl-ACP by MS2 (Doan et al., 2009; Chen et al., 2011) supports a hypothesized secondary pathway yielding aliphatic sporopollenin precursors that could link with the aliphatic α-pyrone units (Figure 1, green shading). Two CYTOCHROME P450 enzymes, CYP703A2 and CYP704B1, essential for exine synthesis, catalyze in-chain and ω-hydroxylation of medium- to long-chain fatty acids (Morant et al., 2007; Dobritsa et al., 2009). These P450 enzymes could act prior to ACOS5 to produce polyhydroxylated polyketides or in the production of hydroxylated aliphatic precursors with MS2 (Figure 1). These highly oxygenated potential sporopollenin precursors are conducive to covalent linkage within the polymer.

**Table 1:** Summary of genes and putative orthologs encoding core enzymes required for sporopollenin biosynthesis.

Arabidopsis gene full name (Gene name)	Putative ortholog	Species	Plant classification	Reference
*CYTOCHROME P450 703A2 (CYP703A2)*	*CYP703A3*	*Oryza sativa*	Angiosperm, monocot	Yang et al., 2014, 2018
*CYTOCHROME P450 704B1 (CYP704B1)*	*MS1 and MS2*	*Brassica napus*	Angiosperm, eudicot	Yi et al., 2010
	*CYP704B2*	*Oryza sativa*	Angiosperm, monocot	Li et al., 2010
	*MS26/CYP704B*	*Triticum aestivum*	Angiosperm, monocot	Singh et al., 2017
*ACYL-COA SYNTHETASE (ACOS5)*	*ACOS5*	*Brassica napus*	Angiosperm, eudicot	Qin et al., 2016
	*ACOS5*	*Nicotiana tobacum*	Angiosperm, eudicot	Wang et al., 2013
	*ACOS12*	*Oryza sativa*	Angiosperm, monocot	Li et al., 2016; Yang et al., 2017
	*ACOS6*	*Physcomitrella patens*	Bryophyte	Li et al., 2019b
*POLYKETIDE SYNTHASE A (PKSA) / LESS ADHESIVE POLLEN 6 (LAP6)*	*PKSA*	*Brassica napus*	Angiosperm, eudicot	Qin et al., 2016
	*PKS1*	*Nicotiana tobacum*	Angiosperm, eudicot	Wang et al., 2013
	*GASCL1* and *GASCL2*	*Gerbera hybrida*	Angiosperm, eudicot	Kontturi et al., 2017
	*PKS1*	*Hypericum perforatum*	Angiosperm, eudicot	Jepson et al., 2014
	*LAP6/PKS1*	*Oryza sativa*	Angiosperm, monocot	Wang et al., 2013; Zou et al., 2017; Shi et al., 2018
	*ASCL*	*Physcomitrella patens*	Bryophyte	Colpitts et al., 2011; Daku et al., 2016
*POLYKETIDE SYNTHASE B (PKSB) / LESS ADHESIVE POLLEN 5 (LAP5)*	*PKSB*	*Brassica napus*	Angiosperm, eudicot	Qin et al., 2016
	*PKS2*	*Oryza sativa*	Angiosperm, monocot	Zhu et al., 2017; Zou et al., 2018
*TETRAKETIDE α-PYRONE REDUCTASE 1 (TKPR1) / DIHYDROFLAVONOL 4-REDUCTASE-LIKE 1 (DRL1)*	*TKPR1*	*Oryza sativa*	Angiosperm, monocot	Wang et al., 2013; Xu et al. 2019
*MALE STERILE 2 (MS2)*	*DPW*	*Oryza sativa*	Angiosperm, monocot	Shi et al., 2011; Wang et al., 2014
	*MS6021*	*Zea mays*	Angiosperm, monocot	Tian et al., 2017
	*MS2*	*Physcomitrella patens*	Bryophyte	Wallace et al., 2015

In addition to a critical role for lipid metabolism, there is support for the inclusion of hydroxylated aromatics in sporopollenin (Guilford et al., 1988; Ahlers et al., 1999; Domínguez et al., 1999; Ahlers et al., 2003; Li et al., 2019a; Lutzke et al., 2020; Xue et al., 2020), although no metabolic genes have been shown to be directly involved in the addition of aromatics to the biopolymer. One of the earliest studies to identify a putative genetic link between phenylpropanoid metabolism and sporopollenin production was in Arabidopsis, in which modulated expression of *FERULIC ACID 5-HYDROXYLASE* (*F5H*) and *CAFFEIC ACID O-METHYLTRANSFERASE* (*COMT*) to form unusual lignin with elevated 5-hydroxyguaiacyl units produced male sterility and abnormal exine (Weng et al., 2010). In rice, the Defective Pollen Wall 2 (DPW2) BAHD acyltransferase catalyzing the transfer of hydroxycinnamoyl moieties to ω-hydroxy fatty acids has been shown to be essential for pollen wall formation (Xu et al., 2017), although it is unclear whether the DPW2 product is directly incorporated into sporopollenin. Further, the expression of Arabidopsis phenylpropanoid pathway genes in the tapetum, alongside *ACOS5*, *PKSA/LAP6*, *PKSB/LAP5*, *TKPR*, *MS2*, *CYP703A2*, and *CYP704B1* supports their involvement in sporopollenin production (Xue et al., 2020).

Over the last decade, the expansion of studies characterizing sporopollenin synthesis-related genes and their encoded enzymes in a variety of species has revealed crucial and largely conserved functionalities in spore/pollen wall formation among land plants. In rice, orthologous genes for each Arabidopsis sporopollenin biosynthesis-related gene have been characterized, including *DPW* (Shi et al., 2011), *CYP703A3* (Yang et al., 2014, 2018), *CYP704B2* (Li et al., 2010), *ACOS12* (Li et al., 2016; Yang et al., 2017), *PKS1*/*LAP6* (Wang et al., 2013; Zou et al., 2017; Shi et al., 2018), *PKS2* (Zhu et al., 2017; Zou et al., 2018), and *TKPR1* (Xu et al., 2019), and exhibit largely redundant gene product activities (Table 1). These findings, together with the characterization of orthologs required for sporopollenin biosynthesis in monocots *Zea mays* (Tian et al., 2017) and *Triticum aestivum* (Singh et al., 2017), and eudicots *Brassica napus* (Yi et al., 2010; Qin et al., 2016), *Nicotiana tobacum* (Wang et al., 2013), *Gerbera hybrida* (Kontturi et al., 2017), and *Hypericum perforatum* (Jepson et al., 2014) support strong conservation of a core sporopollenin metabolic pathway in angiosperms (Table 1). Despite a deficiency in the study of sporopollenin-related orthologs in non-flowering species, characterization of *MS2* (Wallace et al., 2015), *ACOS6* (Li et al., 2019b), and *ASCL* (Colpitts et al., 2011; Daku et al., 2016) in the early emerging moss *Physcomitrella patens* supports substantial overlap in gene product activities, lending further support to the conservation of sporopollenin biosynthetic machinery among land plants (Table 1). Further, ACOS, PKS, and TKPR appear to function cooperatively as a metabolon, forming an ER-localized multienzyme complex in the tapetum of Arabidopsis, *Brassica napus*, and *Nicotiana tobacum* (Lallemand et al., 2013; Wang et al., 2013; Qin et al., 2016), or dually present in the tapetum and locule of rice anthers (Wang et al., 2018; Xu et al., 2019). Altogether, the hypothesis that the synthesis of core sporopollenin constituents involves a highly conserved pathway has gained broad support, reinforcing earlier phylogenetic and genomic findings (Souza et al., 2008; Colpitts et al., 2011; Wang et al., 2013; Gómez et al., 2015; Shi et al., 2015; Zhang et al., 2016) and discoveries made in Arabidopsis. Although understanding of sporopollenin’s chemical composition remains incomplete, molecular genetics have provided a valued mechanism for making informed inferences on sporopollenin’s composition.

## Putative Heterogeneity of Sporopollenin in the Plant Kingdom

Evidence from genetic and biochemical studies indicates polyhydroxylated aliphatic derivatives form major constituents of sporopollenin, which appear to be produced by conserved and ancient biochemical pathways. Yet, the extent of chemical conservation of the biopolymer among land plants remains unclear. With the growth of experimental evidence for sporopollenin-related putative orthologs and their encoded enzymes from diverse plant species (Table 1), data from several studies are challenging the notion of a single definition for sporopollenin. In the case of *ACOS* orthologs, Arabidopsis, rice, tobacco, and canola ACOS enzymes accept similar medium- to long-chain fatty acid substrates, indicating general conservation of enzymatic activities among flowering plants (Souza et al., 2009; Wang et al., 2013; Li et al., 2016; Qin et al., 2016; Yang et al., 2017). Interestingly however, amidst strong conservation of orthologous genes from disparate plant groups, examples of variation in substrate preferences and functional complementation capabilities for select sporopollenin-related enzymes have emerged. Notably, the abnormal exine in Arabidopsis and other dicot sporopollenin biosynthetic mutants commonly manifests as dual abnormalities in rice and other monocots, disrupting exine and anther cuticle formation. The incomplete complementation of the Arabidopsis *acos5* mutant exine phenotype by OsACOS12, driven by the Arabidopsis *ACOS5* promoter, suggests some functional divergence between dicot and monocot ACOS enzymes (Li et al., 2016). In bryophytes, investigations of sporopollenin synthesis-related genes, including *ACOS6* (Li et al., 2019b), *ASCL*, a *PKSA*/*LAP6* ortholog (Colpitts et al., 2011; Daku et al., 2016), and *MS2* in *Physcomitrella patens* (Pp; Wallace et al., 2015), support general conservation of function, with knock-out mutations in each producing defective spore walls. However, complementation analyses using PpACOS6 or PpMS2 driven by their respective Arabidopsis promoters failed to recover *acos5* and *ms2* phenotypes (Wallace et al., 2015; Li et al., 2019b). In support of mixed complementation data, unique substrate preferences for ACOS5 and its orthologs in Physcomitrella and rice suggest sporopollenin fatty acid chain lengths may vary among species (Li et al., 2019b). The breadth of substrates accepted by PpACOS6 also appears reduced relative to AtACOS5 and OsACOS12, suggesting a degree of functional divergence in sporopollenin biosynthetic machinery that may translate into biochemical heterogeneity in the biopolymer across the plant kingdom.

## The Molecular Structure of Sporopollenin

Despite its incredible properties and perhaps because of its resilience, the molecular structure of sporopollenin has not yet been fully elucidated. Recent works, however, report important progress in defining the molecular structure of sporopollenin. Li et al. developed and optimized chemical degradation protocols in association with state-of-the-art NMR techniques to study sporopollenin from the gymnosperm *Pinus rigida* (Li et al., 2019a). With the large amounts of pollen that can be obtained from pine and an optimized thioacidolysis method, sporopollenin was partially solubilized. Analysis of the degradation products by HPLC-UV-MS and NMR detected p-coumaroylated 7-OH-C16 aliphatic chains and the flavonoid naringenin as a minor element. Acetylation of the remaining insoluble fraction allowed partial solubilization, with further NMR analysis identifying mostly polyhydroxylated aliphatic chains, polyvinylacetate (PVA)-like units, flanked at one end by an α-pyrone moiety and crosslinked at the other end through an ester group. These units were found crosslinked with coumaroylated 7-OH-C16 fatty acids through a newly described dioxane moiety (Figure 1). Glycerol-like units, and to a lesser extent naringenin, were linked to PVA-like units through ester bonds. It is suggested that the α-pyrone PVA-like units could be obtained through extension of a hydroxylated fatty acyl precursor with several cycles of malonyl-CoA condensation by type III polyketide synthases (such as PKSA/PKSB) followed by a reduction of the ketone, possibly by TKPR1/DRL1. These units would then be crosslinked with p-coumaroylated 7-OH-C16 aliphatics, flanked on one or both ends by aldehydes, to form the dioxane moiety. In this model, sporopollenin crosslinking involves acetal linkages (forming the dioxane moieties) and ester linkages, having distinct chemical stability. The authors suggest that this chemical linkage heterogeneity explains the superior stability of sporopollenin relative to other biopolymers that are predominantly crosslinked *via* only one linkage type.

In another recent study, Mikhael et al. report novel insights on the molecular structure of sporopollenin from *Lycopodium clavatum* non-sexual spores (Mikhael et al., 2020). Using a combination of TOF-SIMS and CID-MS/MS, MALDI-TOF-MS and CID-TOF/TOF-MS/MS and solid-state NMR, they describe two main units that form the sporopollenin exine in this pteridophyte. The backbone is reported to contain a unique macrocyclic oligomeric monomer of polyhydroxylated aliphatic α-pyrone units, linked through ether bonds, to form a ring-like structure (Figure 1, blue shading). The second unit is composed of polyhydroxylated aliphatic chains with glycerol as a core component, which forms a dendrimer-like structure (Figure 1, green shading) and is reported to be covalently attached by ether bonds to the macrocyclic backbone to form sporopollenin. In contrast to Li et al., an absence of aromaticity was reported for *L. clavatum* sporopollenin. Using infrared spectroscopy, Lutzke et al. report analysis of isolated *Pinus ponderosa* sporopollenin (Lutzke et al., 2020). In agreement with known or hypothesized constituents of sporopollenin, they show that the biopolymer consists of distinct aliphatic and aromatic domains, but in contrast with models proposed by Li et al. and Mikhael et al., α-pyrones were not detected in FTIR spectra. They also report that exine morphology is largely retained after removal of all aromatics, suggesting phenolic compounds are not critical structural components. Similarly, Xue et al. report NMR analysis of sporopollenin from a variety of seed plants, pteridophytes and bryophytes (Xue et al., 2020). Their results indicate the presence of aromatics in all tested sporopollenins, including *Lycopodium clavatum*, contrasting with data presented by Mikhael et al. (2020).

## Cross-Linkages and Assembly of Sporopollenin

To date, no enzyme is known to be involved in the polymerization and deposition of sporopollenin on the microspore surface. It has however been suggested that oxidative polymerization involving ROS takes place, similar to what is known for lignin (Matveyeva et al., 2012; Jacobowitz et al., 2019), and that sporopollenin deposition into an elaborately patterned and sculptured cell wall is guided by a cellulosic (primexine) scaffold and the physico-chemical properties of modulated phases surrounding developing microspores (Scott, 1994; Paxson-Sowders et al., 1997; Gabarayeva et al., 2019; Radja et al., 2019). The application of ROS scavenger to developing moss spores compromised spore wall formation and structural integrity in a dose-dependent manner, suggesting the involvement of ROS and peroxidases in sporopollenin polymerization and/or deposition (Rabbi et al., 2020). In Arabidopsis, tapetum-expressed peroxidases PER9 and PER40 are reported to be mostly involved in tapetal cell wall formation. Primary or secondary effects on pollen wall formation are however also described in the null mutants (Jacobowitz et al., 2019), supporting the oxidative polymerization of sporopollenin.

## Discussion

Through the advancement of tools for solubilizing and fractionating sporopollenin, alongside expansion of studies characterizing sporopollenin biosynthesis in diverse plant species, immense strides toward structurally elucidating sporopollenin and the mechanisms of its biosynthesis have been made. Genetic and biochemical efforts to characterize sporopollenin are converging on similar findings, with extensive overlap in the core subunits and biosynthetic enzyme activities identified, collectively supporting a backbone of covalently coupled polyhydroxylated α-pyrones and hydroxylated aliphatic chains. Although much uncertainty surrounding this complex, resilient organic material remains, advancements in elucidating the molecular structure and biosynthetic mechanisms that form sporopollenin are enabling comparative analyses among land plants and raising new questions about the extent of sporopollenin’s chemical conservation (Xue et al., 2020). Further, mechanisms of assembly, higher-order structure, and the plasticity of the polymer remain largely unexplored topics of basic and applied interest.

Recent biochemical studies reporting polymer analysis describe the presence of hydroxylated, aliphatic α-pyrones in sporopollenin (Li et al., 2019a; Mikhael et al., 2020), upholding composition predictions based on *in vitro* activities of enzymes required for sporopollenin formation. Although biochemical and genetic approaches are forming a more consistent picture of sporopollenin, significant gaps remain between models of sporopollenin proposed by biochemists and what is known of its biosynthetic pathway. In Lycopodium and pine sporopollenin models, aliphatic precursors have in-chain and terminal hydroxyl groups. It is not clear whether the known cytochrome P450 oxygenases (CYP703A2 and CYP704B1) produce these hydroxylations or if other, yet-to-be-discovered oxygenases are involved. In *Pinus rigida*, it is hypothesized that aliphatic precursors bear aldehyde groups at one or both ends to form acetal bonds with the aliphatic α-pyrone. This production would require the action of unknown alcohol dehydrogenases or reductases from alcohol or carboxylic acid precursors, respectively. Similarly, the presence of coumaroyl and naringenin moieties in pine sporopollenin suggests the involvement of not-yet-discovered acyltransferases. Thus, genetic studies have the potential to bridge the remaining gaps in our knowledge of sporopollenin biosynthesis with insights from molecular structure analyses.

The molecular structure of sporopollenin has been a mystery for decades with numerous studies only providing glimpses of functional groups or fragments of the polymer constituents, such as lipids or phenol rings. It is now suggested that some earlier discoveries identified technical artifacts arising from the harsh conditions used for sporopollenin analysis (Gonzalez-Cruz et al., 2018; Mikhael et al., 2020). Using different models, recent studies present significant progress in understanding sporopollenin’s molecular composition. Li et al. describe a principal structure of hydroxylated aliphatic α-pyrone units linked by coumaroylated 7-OH-C16 aliphatic in pine. Lutzke et al. report aliphatic and aromatic domains but no polyketide-derived elements in pine sporopollenin. Mikhael et al. describe a macrocyclic backbone structure composed of hydroxylated aliphatic α-pyrone units linked by dendrimer-like, hydroxylated fatty acid units in *L. clavatum* with no aromatics, while Xue et al. report the presence of aromatics for the same plant. Thus, despite recent advances, inconsistencies remain in the available sporopollenin molecular structure data, depending on the techniques used or organism studied.

General consensus describes sporopollenin with a main polyhydroxylated aliphatic component and polyketide-derived aliphatic α-pyrone elements. This emerging picture reconciles for the first time the work of biochemists with that of geneticists that described the involvement of fatty acid oxygenase cytochrome P450s, polyketide synthases, and polyketide reductases in the biosynthesis of sporopollenin. Genetic studies have uncovered remarkable conservation of the core sporopollenin biosynthetic pathway in the plant kingdom. However, differences found recently question the extent of sporopollenin chemical conservation between evolutionary clusters. It is possible that the backbone of sporopollenin between plant clusters is conserved but differs in the way the units are assembled or “decorated” with phenolic-derived elements. Continued efforts to chemically and structurally characterize sporopollenin from flowering and early emerging plant species will help to resolve current discrepancies and provide evolutionary context for resolving sporopollenin’s compositional homogeneity within the plant kingdom.

The recent acceleration of long-term efforts to uncover the mysteries of sporopollenin, particularly with solid-state methods, has provided novel understanding of the molecular features of the biopolymer that confer extreme stability. Harnessing the resilience of sporopollenin offers diverse opportunities for synthetic material design in medical science, space exploration, and environmental remediation. Further, the use of sporopollenin-based microparticles as a vector for oral vaccines and a range of macromolecules offers potential for sustainable applications of this abundant and stable biomaterial.

## Author Contributions

All authors listed have made a substantial, direct and intellectual contribution to the work, and approved it for publication.

## Conflict of Interest

The authors declare that the research was conducted in the absence of any commercial or financial relationships that could be construed as a potential conflict of interest.

## Publisher’s Note

All claims expressed in this article are solely those of the authors and do not necessarily represent those of their affiliated organizations, or those of the publisher, the editors and the reviewers. Any product that may be evaluated in this article, or claim that may be made by its manufacturer, is not guaranteed or endorsed by the publisher.
